# The TRPV1 ion channel regulates thymocyte differentiation by modulating autophagy and proteasome activity

**DOI:** 10.18632/oncotarget.21798

**Published:** 2017-10-11

**Authors:** Consuelo Amantini, Valerio Farfariello, Claudio Cardinali, Maria Beatrice Morelli, Oliviero Marinelli, Massimo Nabissi, Matteo Santoni, Laura Bonfili, Valentina Cecarini, Anna Maria Eleuteri, Giorgio Santoni

**Affiliations:** ^1^ School of Biosciences and Veterinary Medicine, University of Camerino, Camerino, Italy; ^2^ University of Lille, INSERM U1003 - PHYCEL - Physiologie Cellulaire, Lille, France; ^3^ School of Pharmacy, Experimental Medicine Section, University of Camerino, Camerino, Italy; ^4^ Department of Molecular Medicine, Sapienza University, Rome, Italy

**Keywords:** ER stress, capsaicin, TRPV1, TRPV1 KO mice, autophagy, Immunology and Microbiology Section, Immune response, Immunity

## Abstract

Autophagy and the ubiquitin-proteasome system (UPS) control thymus cell homeostasis under resting and endoplasmic reticulum (ER) stress conditions. Several evidence support a cross-talk between UPS and autophagy; abrogation of UPS responses stimulates autophagy, and vice versa the inhibition of autophagy alters the UPS functions.

Herein, we found that TRPV1 activation induces ER stress, proteasome dysfunction and autophagy in thymocytes by modulating the expression of UPR-related genes. The TRPV1-mediated autophagy prevents the UPR activation by inhibiting BiP, Grp94 and ERp57 chaperone protein expression.

Thymocytes from TRPV1 KO mice display both autophagy and proteasome dysfunctions, resulting in increased apoptotic cells and reduced total DP thymocyte number.

In addition, positive selection of thymocytes triggered by anti-TCRβ/CD2 Ab-mediated costimulation induces apoptosis in thymocytes from TRPV1 KO as compared with WT mice. Stimulation of TRPV1 KO thymocytes with anti-TCRβ/CD2 mAbs modulates the expression of CD4 antigen on purified DP thymocytes, with reduced number of mature, single positive (SP) CD4 and increased number of immature SP CD4^low^ and DP CD4^low^CD8^+^ thymocytes, further supporting the intrinsic role of TRPV1 in T cell maturation. Finally, a reduction in CD8^+^ and CD4^+^ T cells is evidenced in the peripheral blood and spleen of TRPV1 KO, as compared with WT mice. Therapeutic strategy by restraining or stimulating the TRPV1 expression and functions in thymocytes might represent a new pharmacological tool in the regulation of different inflammatory T cell responses.

## INTRODUCTION

Autophagy and the ubiquitin-proteasome system (UPS) constitute the major intracellular degradation pathways controlling cellular homeostasis under normal conditions and cellular stress [1]. Autophagy is a cellular adaptive response to stress conditions, such as nutrient, growth factor deprivation or oxidative stress. This process is mainly involved in the degradation of long-lived proteins and excess/damaged organelles [2]. The UPS represents the principal degradation mechanism for short-lived proteins labeled with ubiquitin [3]. Unfolded or misfolded proteins are tagged for degradation *via* endoplasmic reticulum-associated degradation (ERAD). Continued accumulation of incorrectly folded proteins triggers the unfolded protein response (UPR) in the attempt to resolve ER stress and reestablish the folding homeostasis [4-6]. Mild to moderate ER stress induces autophagy as a compensatory cell survival mechanism by relieving proteasome inhibitor-induced ER stress, whereas severe or chronically prolonged ER stress deteriorates cellular functions with a switch from an adaption program to apoptotic cell death [7, 8]. In cases in which mild ER stress activates all UPR sensors, survival is favored as a consequence of increased instability of the mRNAs and proteins that promote apoptosis compared to those that facilitate adaptation and autophagic survival [8]. Several evidence support the existence of an interplay between the UPS and autophagy [9]: inhibition of UPS often induces autophagy whilst inhibition of autophagy alters the UPS function [1].

Autophagy is vital for thymocytes during stress conditions such as starvation, activation, growth and proliferation to provide cells with essential metabolic intermediates. This process is involved in T cell thymic development and the thymus exhibits a considerable high amount of constitutive basal autophagy compared to other tissues. In thymic antigen presenting cells (APCs), intracellular peptides can be presented on MHC class II through autophagy [10].

Members of the transient receptor potential (TRP) ion channel family have been shown to mediate cellular Ca^2+^ homeostasis, initiate ER stress and stimulate UPR and apoptosis, or autophagic survival in normal and neoplastic cells [11]. In this regard, TRPV1 is a cation channels expressed on thymocytes as well as on naïve and effector CD4^+^ T lymphocytes and Jurkat T cell leukemia [12, 13], and a contribute of TRPV1 in TCR-induced Ca^2+^ influx and proper downstream TCR signaling leading to T cell activation has been recently reported [14]. Previously, we have reported that the TRPV1 agonist, capsaicin (CPS) modulates T cell differentiation and functions by regulating the apoptosis of distinct thymocyte subpopulations in rats [12]. Moreover, the presence of an interplay between autophagic survival and apoptotic cell death in response to stress signals has been demonstrated in the mouse thymus [15, 16]. Triggering of TRPV1 by the specific agonist CPS, induces autophagic survival in mouse DP^dull^ thymocytes. TRPV1-induced autophagy is Atg4C- and Atg6-dependent and, requires [Ca^2+^]_i_ increase and reactive oxygen species (ROS) generation that induces Atg4C protein oxidation resulting in AMPK activation. In addition, the inhibition of TRPV1-mediated autophagy by the 3-MA autophagic inhibitor decreases Atg4C, Bcl-XL, Irgm1 and Beclin-1 expression and induces caspase-3-dependent apoptosis of DP^dull^ thymocytes [15].

At present, the molecular mechanism involved in thymus maturation by regulating thymocyte fate has been only partially investigated. Thus, the aim of the present work was to evaluate the role of TRPV1 in the cross-talk between ER stress, proteasome and autophagy responses, resulting in thymocyte survival or death in steady state and during thymic maturation.

## RESULTS

### CPS inhibits cellular proteasome function in a TRPV1-dependent manner

**Figure 1:**
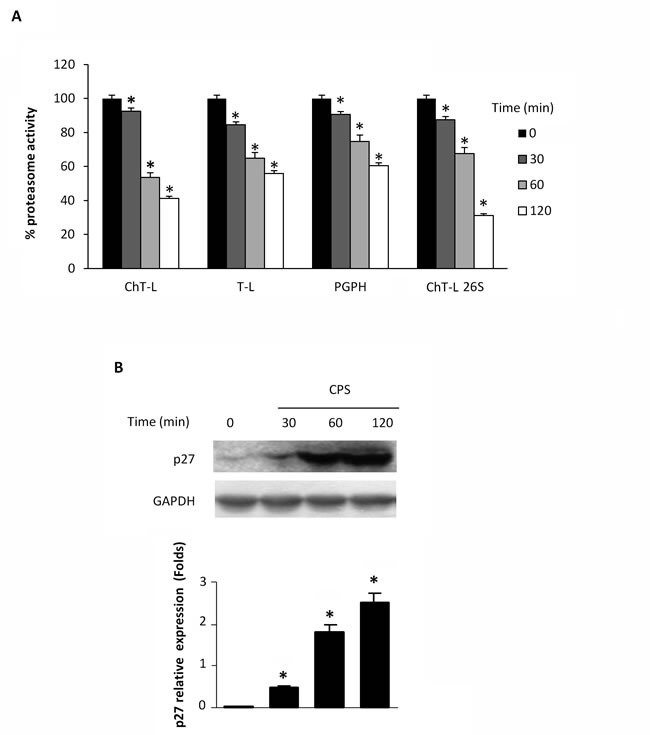
CPS inhibits cellular proteasome activity **A.** WT thymocytes were subjected to enzymatic proteasome activity assay after 30, 60 and 120 minutes of CPS treatment. Data, shown as percentage of proteasome activity, are from one representative experiment out of three separate experiments. Error bars are relative to three replicates. *p < 0.01 *vs* untreated cells. **B.** Western blot analysis and densitometric quantification of p27 protein levels in thymocytes treated for up to 120 minutes with CPS. p27 densitometry values were normalized to GAPDH used as loading control. Blots are representative of one of three separate experiments. *p < 0.01 treated *vs* untreated cells.

**Figure 2:**
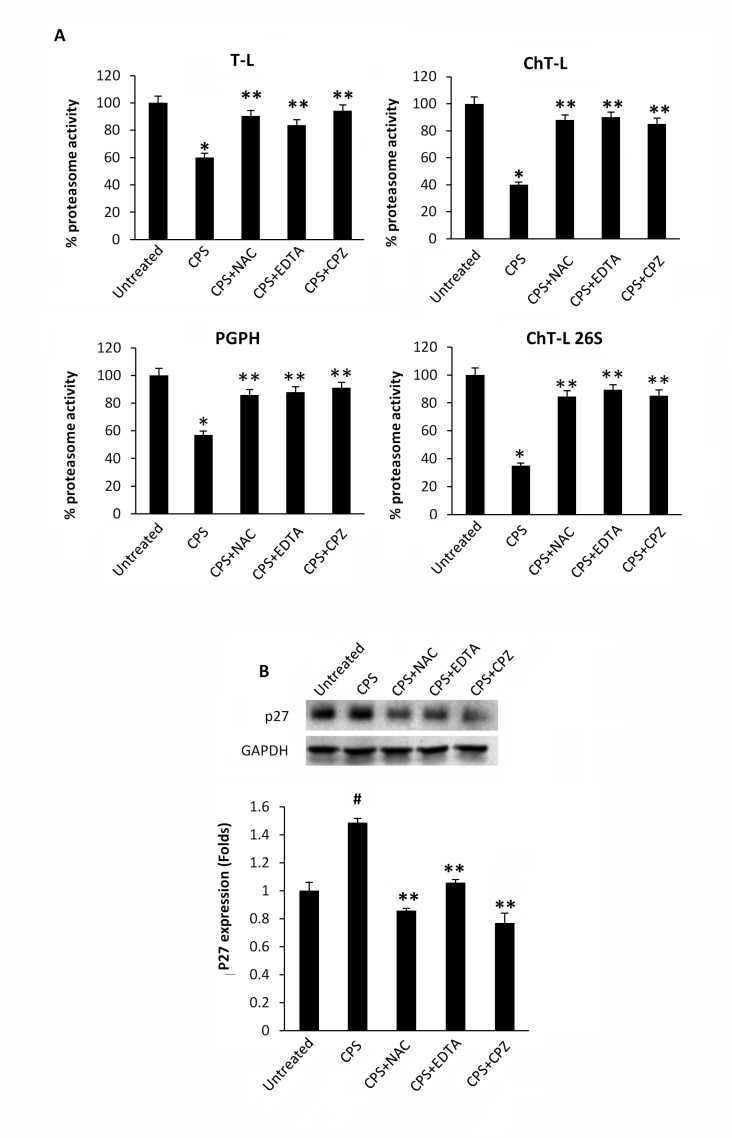
CPS-mediated inhibition of enzymatic proteasome activity is ROS-, Ca^2+^- and TRPV1- dependent **A.** Enzymatic proteasome activities were assessed in WT thymocytes pre-treated with NAC, EDTA and CPZ for 60 minutes and then treated with CPS for 120 minutes. Data, shown as percentage of proteasome activity, are from one representative experiment out of three separate experiments. Error bars are relative to three replicates. *p < 0.01 *vs* untreated cells; **p < 0.01 *vs* CPS-treated cells. **B.** Western blot analysis and densitometric quantification of p27 protein levels in thymocytes treated as above described. p27 densitometric values were normalized to GAPDH used as loading control. Blots are representative of one of three separate experiments. #*p* < 0.01 *vs* untreated; **p < 0.01 *vs* CPS-treated cells.

Lysates from CPS-treated thymocytes were analyzed for proteasome activity. As shown in Figure 1A, exposure of thymocytes to the TRPV1 agonist, CPS resulted in a time-dependent decrease of ChT-L, T-L, PGPH and 26S proteasome ChT-L activities. To further confirm the effect of CPS on cellular proteasome, the expression level of the proteasome target protein p27 was measured by immunoblot analysis. Figure 1B demonstrates that TRPV1-mediated proteasome inhibition correlates with increased levels of p27 in thymocytes. Then, the involvement of ROS generation and the increase of [Ca^2+^]_i_ in TRPV1-mediated inhibition of the proteasome activity was evaluated by using the ROS scavenger NAC, the Ca^2+^ blocker EDTA and the specific TRPV1 antagonist, capsazepine (CPZ). NAC, EDTA and CPZ completely reverted the inhibition of proteasome functionality (Figure 2A) and the p27 accumulation (Figure 2B) induced by CPS in a TRPV1-mediated manner after 2 hours of treatment.

### The ER stress inhibitor 4-PBA abrogates the CPS-induced autophagy promoting thymocyte apoptosis

**Figure 3:**
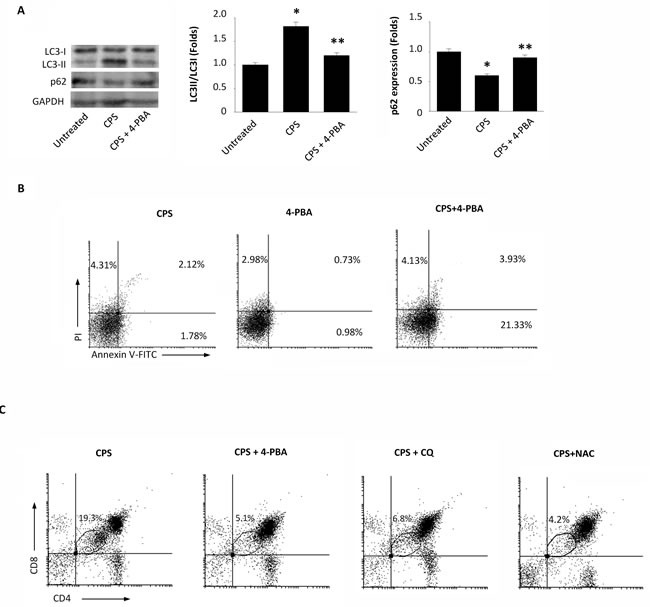
The 4-PBA abrogates the CPS-induced autophagy and reduces the DP^dull^ thymocyte subpopulation **A.** Western blot analysis of LC3 and p62 protein in WT thymocytes pre-treated with 4-PBA for 60 minutes and then treated for 120 minutes with CPS. The ratio of LC3 II/I was calculated from densitometric data. p62 densitometric values were normalized to GAPDH used as loading control. Blots are representative of one of three separate experiments. *p < 0.01 *vs* untreated cells, **p < 0.01 *vs* CPS-treated cells. **B.** Flow cytometric analysis was performed by Annexin V-FITC and PI double-staining of WT thymocytes, pre-treated with 4-PBA for 60 minutes and then treated for 120 minutes with CPS. Data represent the percentage of PI and/or Annexin V positive cells. One representative out of three independent experiments is shown. **C.** Thymocytes, pre-treated with 4-PBA, CQ or NAC for 60 minutes and then treated for 120 minutes with CPS, were stained with anti-CD4-PE and anti-CD8-Cy5 mAbs and analyzed by FACS. The black gate indicates the DP^dull^ subpopulation. One representative out of three independent experiments is shown.

Previously, it has been demonstrated that proteasome inhibition activates autophagy to purge polyubiquitinated protein aggregates with the aim to alleviate ER stress and the UPR [17]. Starting from these results and taking into account our recent data demonstrating that TRPV1 activation by CPS triggers autophagy counteracting apoptotic cell death of DP^dull^ thymocytes [15], we decided to evaluate whether autophagy could occur as a consequence of CPS-induced proteasome inhibition and ER stress. To this aim, thymocytes were treated with CPS in combination with 4-PBA, a small chemical chaperone that interacts with the hydrophobic domains of misfolded proteins, preventing their aggregation and supporting the correct protein folding [18]. The autophagy pathway was evaluated by immunoblotting using specific anti-LC3 and -p62 antibodies. In fact, during autophagy, the cytosolic form of LC3 (LC3I) binds phosphatidylethanolamine to form LC3-phosphatidylethanolamine conjugate (LC3II), which is recruited to autophagosomal membranes [19], whereas the receptor for cargo p62 is degraded [20]. Our results demonstrated that TRPV1-induced autophagy depends on ER stress, as 4-PBA completely inhibited LC3II formation and p62 degradation (Figure 3A).

In addition, Annexin V/PI or CD4/CD8α staining followed by FACS analysis revealed that 4-PBA in combination with CPS increases the percentage of Annexin V^+^PI^-^ early (21.33%) and Annexin V^+^PI^+^ late (3.93%) thymocytes (Figure 3B), and reduces the TRPV1-dependent increase of DP^dull^ thymocytes (Figure 3C). Similar results were obtained in CPS-treated thymocytes pretreated with the autophagic inhibitor CQ or with the ROS scavenger NAC (Figure 3C). In addition, no major changes in the expression levels of the activation markers CD25 and CD69 were found on CPS-treated as compared with untreated thymocytes (Supplementary Figure 1).

### TRPV1 activation modulates the expression of UPR-related genes

UPR activation, upon proteasome dysfunction and ER stress, is a well-documented process [21-23]. Based on these observations, we investigated the effect of the TRPV1 agonist CPS on the expression of UPR components in thymocytes at 1 hour after treatment by RT-PCR array. In particular, the comparison of gene expression levels of CPS-treated with untreated thymocytes (Table 1), demonstrates that Hspa5 (Bip) and Hspa1 (heat shock proteins involved in protein folding), Dnajb9 (regulator of the ATPase activity of 70 kDa heat shock proteins), Bax (pro-apoptotic protein), Xbp1 (transcription factor promoting the expression of genes involved in protein degradation), Insig1 (involved in cell growth) Mbtps2 (regulators of oxidative stress susceptibility) and the Rpn1 (part of the subunit of the 26S proteasome) are down-regulated, whereas Derlin-1 (functional component of ERAD and autophagy), Derlin-2 (functional component of ERAD) and Ero1lb (oxidoreductase involved in protein folding) are up-regulated. Overall, these findings demonstrate that CPS inhibits the expression of UPR and apoptosis genes and enhances that involved in modulation of ERAD and autophagy processes.

**Table 1:** CPS induces UPS gene expression changes.

Ref Seq	Symbol	Description	Fold change CPS-treated vs untreated
NM_007527	Bax	Bcl2-associated X protein	-2.04±0.03*
NM_013760	Dnajb9	Dnaj (Hsp40) homolog, subfamily B, member 9	-2.09±0.08*
NM_022310	Hspa5	Heat shock protein 5	-2.79±0.02*
NM_153526	Insig1	Insulin induced gene 1	-3.31±0.02*
NM_172307	Mbtps2	Membrane-bound transcription factor peptidase, site 2	-2.10±0.02*
NM_133933	Rpn1	Ribophorin 1	-2.40±0.04*
NM_013842	Xbp1	X-box binding protein 1	-2.67±0.05*
NM_013558	Hspa1l	Heat shock protein 1-like	-2.27±0.43*
NM_024207	Derl1	Der1-like domain family, member 1	+2.65±0.03*
NM_033562	Derl2	Der1-like domain family, member 2	+2.09±0.02*
NM_026184	Ero1lb	ERO1-like beta (S.Cerevisiae)	+2.10±0.04*

RT-PCR array in mRNA samples from thymocytes untreated and treated with CPS for 60 min. The expression levels were normalized using the housekeeping gene GAPDH and calculated by the ∆∆Ct method. Data, shown as fold change, represent the mean ± SD of three experiments. Untreated thymocyte mRNA was used as calibrator. *p < 0.01 vs untreated cells.

### The autophagy and ER stress inhibitors revert the TRPV1-mediated UPR down-regulation in CPS-treated thymocytes

**Figure 4:**
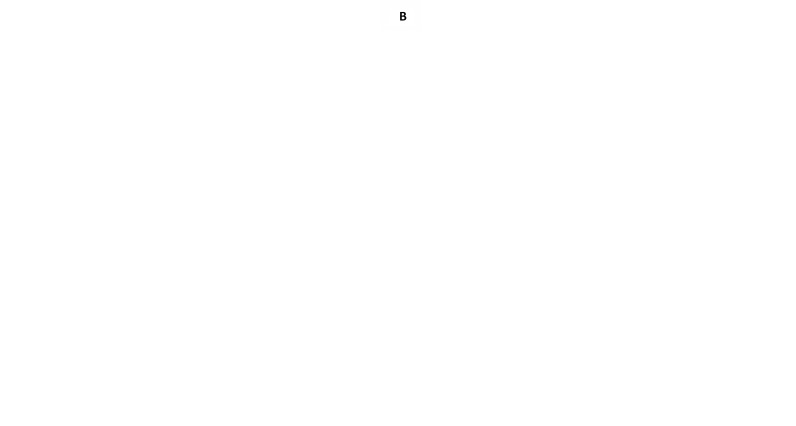
CPS reduces UPR protein levels, and autophagy reverts CPS-induced inhibitory effects **A.** Western blot analysis and densitometric quantification of Grp94, BiP and ERp57 protein levels in WT thymocytes pre-treated with CQ and 4PBA for 1h and then treated for up to 4h with CPS. Blots are representative of one of three separate experiments. **B.** Densitometric quantification of Grp94, BiP and ERp57 protein levels in WT thymocytes treated with CPS for different times. Densitometric values were normalized to GAPDH used as loading control and represent the mean ± SEM of three separate experiments. *p < 0.01 *vs* untreated cells. **C.** Densitometric quantification of Grp94, BiP and ERp57 protein levels in thymocytes pre-treated with CQ and 4PBA and then treated with CPS. *p < 0.01 *vs* CPS-treated cells. Densitometric values were normalized to GAPDH used as loading control and represent the mean ± SEM of three separate experiments.

The TRPV1-induced changes in the UPR gene expression (Table 1) prompted us to investigate by western blot analysis, the expression of UPR proteins such as BiP, Grp94 and ERp57 chaperones, in thymocytes treated for different times (1, 2 and 4h) with CPS. We found that CPS decreases in a time-dependent manner, BiP, Grp94 and ERp57 protein expression (Figure 4A, 4B). Pretreatment of thymocytes with CQ or 4-PBA, before CPS exposure, completely reverted the CPS-induced UPR protein reduction, in a time-dependent manner, allowing their accumulation (Figure 4A, 4C), suggesting that CPS-stimulated autophagy prevents the ER stress-induced UPR.

### Thymocytes from TRPV1 KO mice display both autophagy and proteasome dysfunctions

**Figure 5:**
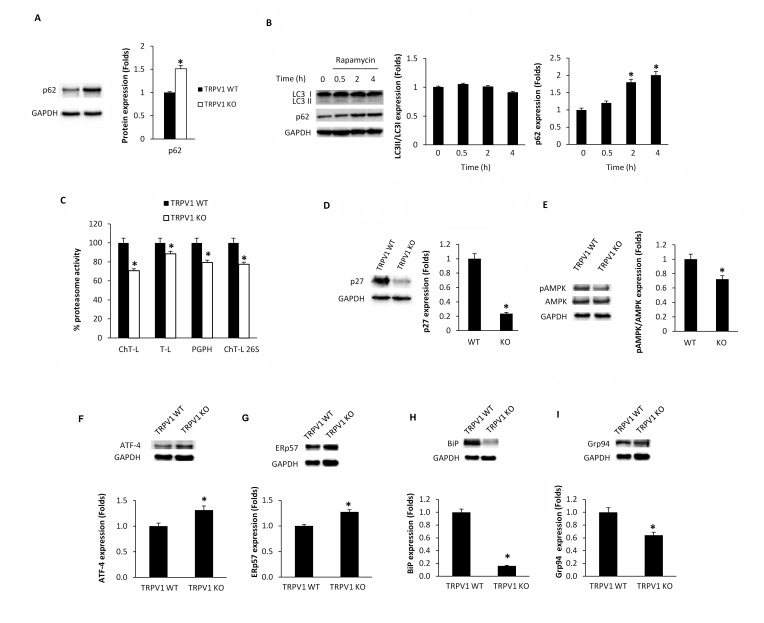
Knock-out of TRPV1 gene affects autophagy, proteasome and UPR protein expression in thymocytes **A.** Western blot analysis and densitometric quantification of basal p62 protein levels in WT and TRPV1 KO thymocytes. Densitometric values were normalized to GAPDH used as loading control. Blots are representative of one of three separate experiments, *p < 0.01 *vs* WT thymocytes. **B.** Western blot analysis and densitometric quantification of LC3 and p62 protein levels in TRPV1 KO thymocytes treated for up to 4 h with rapamycin. Densitometric values were normalized to GAPDH used as loading control. Blots are representative of one of three separate experiments, *p < 0.01 *vs* CPS-treated cells for 0.5h or untreated cells. **C.** Thymocytes from WT and TRPV1 KO mice were subjected to enzymatic proteasome activity assay. Data shown as percentage of inhibition are representative of one of three separate experiments, *p < 0.01 TRPV1 KO *vs* WT thymocytes. D-I. Western blot analysis and densitometric quantification of p27 **D.**, pAMPK/AMPK **E.**, ATF-4 **F.**, ERp57 **G.**, Bip **H.** and Grp94 **I.** protein levels in WT and TRPV1 KO thymocytes. Densitometric values were normalized to GAPDH used as loading control. Blots are representative of one of three separate experiments, *p < 0.01 *vs* WT thymocytes.

Since a role for TRPV1 in CPS-induced autophagy in thymocytes was found [15], we evaluated the consequence of TRPV1 genetic deletion on thymic autophagy. Our results showed that TRPV1 KO thymocytes express higher basal levels of p62 protein compared with WT cells (Figure 5A). Moreover, differently from WT thymocytes [15], TRPV1 KO thymocytes failed to activate the autophagy when stimulated with the autophagy inducer rapamycin (Figure 5B). Since the increase of p62 protein levels and the autophagic dysfunction result in the inhibition of proteasome [24, 25], the proteasome function was evaluated in TRPV1 KO and WT thymocytes. A decrease in the ChT-L, T-L, PGPH and 26S proteasome ChT-L activities (Figure 5C), a reduction of p27 protein and AMPK phosphorylation levels (Figure 5D and 5E) were found in TRPV1 KO thymocytes compared with WT cells. In addition, the apoptotic regulator Atf4 transcription factor (Figure 5F) and ERp57 (Figure 5G) protein levels were up regulated whereas BiP (Figure 5H) and Grp94 (Figure 5I) UPR proteins were reduced in TRPV1 KO compared with WT thymocytes. Overall, these findings demonstrate that the expression of TRPV1 in thymocytes is crucial in regulating autophagy, apoptosis and UPR activity.

### Loss of TRPV1 increases the apoptosis, reduces the total thymocyte number and affectes thymocyte distribution

**Figure 6:**
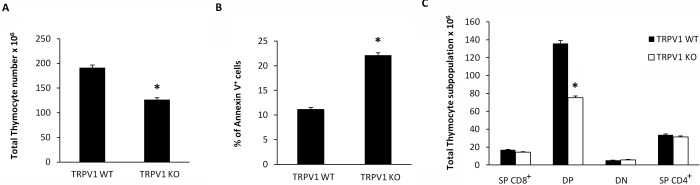
Increased basal levels of apoptotic thymocytes and reduced total thymocyte number in TRPV1 KO mice Freshly purified thymocytes from WT and KO mice were counted using trypan blue solution **A.**, or stained with Annexin-V FITC and analyzed by FACS analysis **B.** Data are the mean ± SEM of three separate experiments, *p < 0.01 *vs* WT thymocytes. **C.** From the total number of cells per thymus, the relative number of cells in each CD4/CD8 subset was determined staining TRPV1 KO and WT thymocytes with anti-CD4-PE and anti-CD8-Cy5 mAbs and using FACS analysis. Data are the mean ± SEM of three separate experiments *p < 0.01 TRPV1 KO *vs* WT thymocytes.

Then we evaluated whether knockout of TRPV1 gene could affect thymocyte number and distribution. To this purpose, freshly isolated thymocytes from TRPV1 KO and WT mice were counted and then labeled with Annexin V. Our findings show a reduction in total thymocyte count (Figure 6A), that is associated with a parallel increase in the percentage of Annexin V^+^ thymocytes (Figure 6B). In addition, knockout of TRPV1 affected thymocyte cell distribution. Thus, a marked reduction of total DP thymocyte number (Figure 6C) was observed in the thymus from TRPV1 KO respect to WT mice.

### Positive selection is impaired in TRPV1 KO thymocytes

**Figure 7:**
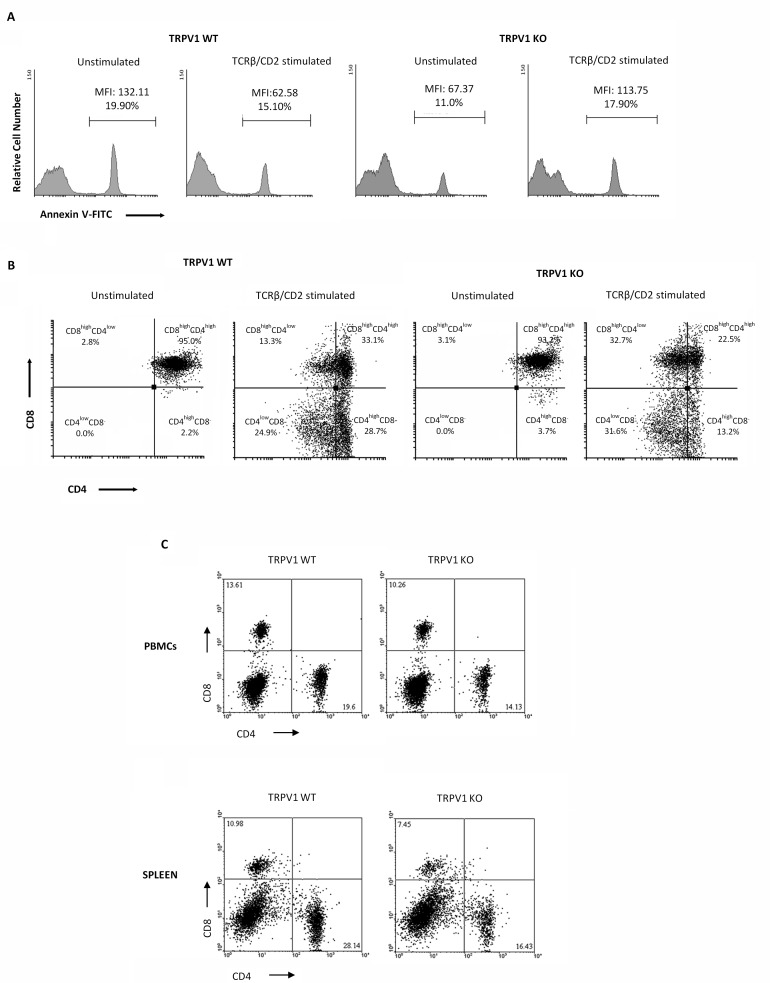
Knock-out of TRPV1 gene impairs positive thymic selection and affects peripheral blood and spleen CD4^+^ and CD8^+^ T cell numbers **A.** Thymocytes from WT and KO mice, stimulated with anti-TCRβ plus anti-CD2 mAbs after 18h of culture and 12h of recovery, were analyzed by FACS analysis. One representative out of three independent experiments is shown. **B.** Purified DP thymocytes from TRPV1 KO and WT mice stimulated with TCRβ plus CD2 mAbs for 18h, followed by 12h recovery, were stained with anti-CD4-PE and anti-CD8-Cy5 mAbs and analyzed by FACS. One representative out of three independent experiments is shown. **C.** Blood and spleen PBMC from WT and KO thymocytes were stained with anti-CD4-PE and anti-CD8-Cy5 mAbs and analyzed by FACS. One representative out of three independent experiments is shown in each panel.

We have previously reported the role of TRPV1 in thymocyte differentiation by regulating the crosstalk between autophagic survival and apoptotic cell death [15]. To understand the contribution of TRPV1 in thymocyte maturation, we mimicked positive selection by stimulating TRPV1 KO and WT thymocytes for 18h with anti-TCRβ plus anti-CD2 mAbs and allowing recovery for 12h [26]. Since positive selection is an essential step for thymocyte survival and TCRβ/CD2 coengagment promotes anti-apoptotic signals [27], we evaluated the extent of apoptotic cell death in un-stimulated and stimulated TRPV1 KO and WT thymocytes by cytofluorimetric analysis. As expected, positive selection promotes cell survival in WT thymocytes, as indicated by the reduced percentage of Annexin V^+^ cells. On the contrary, an impairment of positive selection was observed in TRPV1 KO thymocytes as shown by the increased frequency of apoptotic cells (Figure 7A). These results prompted us to evaluate the outcome of positive selection, in highly purified ( > 97%) TRPV1 KO and WT CD4^+^CD8^+^ DP thymocytes, in response to TCRβ plus CD2 stimulation. Since previous results showed that the positive selection of DP cells promotes the maturation of CD4^+^ thymocytes [26], we performed cytofluorimetric analysis to assess the expression of CD4 and CD8 co-receptors. We found that the maturation process is promoted in both TRPV1 KO and WT thymocytes, as revealed by the modulation of CD4 expression; however, in stimulated TRPV1 KO DP thymocytes, a reduced number of total SP CD4 thymocytes (44.8%) was observed with respect to WT thymocytes (53.6%) (Figure 7B). In addition, CD4 expression levels on mature SP cells was lower in TRPV1 KO (CD4^high^ 13.2% and CD4^low^ 31.6%) with respect to WT thymocytes (CD4^high^ 28.7% and CD4^low^ 24.9%) (Figure 7B). Finally, stimulation of positive selection also induced a marked increase in the percentage of immature CD4^low^CD8^+^ DP subpopulation in TRPV1 KO (32.7%) as compared with WT thymocytes (13.3%) (Figure 7B).

Based on these findings, demonstrating that the absence of TRPV1 increases the basal level of apoptotic thymocytes and impairs thymic positive selection, we finally evaluated the distribution of T lymphocytes in the peripheral blood and spleen. PBMC and splenic lymphocytes from TRPV1 KO and WT control mice were purified by Lympholyte density gradient, stained with anti-mouse CD4 and CD8 mAbs and evaluated by cytofluorimetric and FACS analysis. We observed that the percentage of mature CD8^+^ and CD4^+^ T cells in the peripheral blood and spleen was significantly lower in TRPV1 KO *vs* WT mice (Figure 7C) suggesting that the defect in T cell numbers in the periphery of TRPV1 deficient mice may be the result of the impairment of their maturation in the thymus.

## DISCUSSION

Several evidence support the existence of a cross-talk between UPS and autophagy [9, 28]. Proteasome inhibitors induce autophagy by triggering ER stress [17]; conversely, suppression of both proteasome and autophagy has been shown to enhance apoptotic cell death [29].

The specific TRPV1 agonist CPS has been found to inhibit the proteasome [30, 31] and trigger autophagy [15, 32, 33], however, the relationship between these degradation systems in thymocytes and the role of TRPV1 have not been addressed so far.

Herein, we found that TRPV1-dependent autophagy occurs as a consequence of proteasome inhibition and ER stress triggered by TRPV1. Reduced 20 and 26S proteasome activities and increased p27 expression were found in CPS-treated thymocytes, and this effect was TRPV1-, ROS- and [Ca^2+^]_i_ rise-dependent.

In fact, pretreatment of thymocytes with the 4-PBA that reduces ER stress [18, 34, 35] before the addition of CPS, inhibited LC3II generation and p62 degradation, increased the percentage of AnnexinV^+^ apoptotic cells and completely reverted the CPS-induced increase of DP^dull^ thymocytes, similarly to the autophagic inhibitor CQ. TRPV1 triggering by CPS resulted in inhibition of UPR and apoptosis as well as increase of ERAD and autophagic gene expression. Since a role of Ero1lb in setting ER redox potential and proper ER folding activity [36] and CPS in disrupting redox state by promoting ROS generation [37], the increased Ero1lb mRNA levels we found in CPS treated thymocytes could be the result of CPS-induced TRPV1-mediated ROS generation [15] altering ER redox homeostasis. In addition, CPS exposure triggers derlin-1 mRNA overexpression; although the role of derlin-1 in the protection of thymocytes from apoptosis is still unknown, knock-down of derlin-1 by abrogating p62 degradation, results in blockage of autophagy flux [38].

In CPS-treated thymocytes, reduction of BiP, Grp94 UPR protein levels, as results of UPR inhibition, was completely reverted by pretreatment with 4-PBA or CQ treatment, ER stress and autophagy inhibitors, respectively, strongly suggesting that CPS-induced autophagy is a TRPV1-dependent ER-stress-stimulated process.

The contribution of TRPV1 in the autophagy and proteasome cross-talk was also evaluated in thymocytes from TRPV1 KO mice. Genetic deletion of TRPV1 results in increased basal levels of p62 and in a defective autophagic response to the mTOR inhibitor and autophagy inducer rapamycin. The p62/SQSTM1 is a multifunctional protein selectively degraded by autophagy but not by UPS [20, 38] that accumulates when autophagy function is inhibited [39, 40]. The p62 accumulation during autophagy inhibition appears to be responsible for the defective UPS function, as p62 silencing attenuates the accumulation of proteasome substrates caused by autophagy inhibition, and the pharmacological inhibition of the proteasome increases p62 expression [41, 42]. In this view, impaired autophagy due to TRPV1 genetic deletion by inducing p62 accumulation reduces proteasome functions. The decreased proteasome activity observed in TRPV1 KO thymocytes was associated with loss of AMPK activity and a decreased p27 expression compared with WT thymocytes [15]. Inhibition of AMPK by compound C or by shRNA-mediated depletion of LKB reduces activation of autophagy by rapamycin, and shRNA-mediated depletion of p27 inhibits rapamycin-induced autophagy [43]. Thus, the AMPK/p27 pathway deregulation observed in TRPV1 KO thymocytes, exacerbates the severity of the autophagy/proteasome defects, further promoting the reduction of DP thymocyte number.

Our results in TRPV1 KO thymocytes, also provide evidence of a reduction of BiP and Grp94 UPR proteins and increase of ERp57 levels. Knock-down or inhibition of ERp57 resulted in decreased death-inducing signaling complex and caspase activity [44]. On the contrary, autophagy failure, induced by compromised UPR, has been found to increase the ERp57 expression in beta cell islets from Atg7-deficient mice [45]. Thus, the reduction of ERp57 in CPS-treated WT thymocytes may be the result of enhanced autophagic survival, as well as the increase of ERp57 level in TRPV1 KO thymocytes could promote apoptotic death caused by autophagic failure.

In the same view, Atf4 induction by prolonged stress exposure triggers apoptosis in cancer cells [46]; so, the enhancement of Atf4 protein expression evidenced in TRPV1 KO thymocytes, may lead to increased susceptibility of thymocytes to apoptotic stimuli.

In addition, as result of double defects in autophagic and proteasome degradation systems, increased levels of apoptotic thymocytes associated with a reduction of total and DP thymocyte number was found in TRPV1 KO mice. Accordingly, the proteasome inhibitor Bortezomib, results in a dramatic decrease of thymocytes by inducing ER stress-apoptosis [47].

Proteasome inhibition has been also found to interfere with thymic selection, and thereby to influence the survival of DP and SP thymocytes [48, 49]. Differentiation of immature DP thymocytes into mature SP CD4^+^ and CD8^+^ T cells is referred as positive selection [26, 50], and requires physical contact with thymic cortical epithelium. Indeed, TCRβ and CD2 co-engagement by increasing survival signals [27] promotes a differentiation pathway resulting in generation of SP CD4^+^ T cells from DP thymocytes [26]. This pathway requires two distinct steps in which expression of both co-receptors CD4 and CD8 is reduced (CD4^low^CD8^low^, DP^dull^), followed by selective re-expression of CD4 with the CD4^low^ cells representing the immediate precursor of mature CD4^+^ cells. In this regard, our findings add new straightforward data on the role of TRPV1 in thymocyte maturation. In fact, its absence is responsible for the increased percentage of apoptotic cells after TCR engagement, likely due to the loss of the survival signals that normally characterize the positive selection. The positive selection stimulates the maturation of DP thymocytes in SP CD4^+^ cells both in TRPV1 KO and WT cells; whereas, the impaired TRPV1 signaling in TCRβ/CD2-stimulated thymocytes markedly reduced the percentage of mature SP CD4^+^ thymocytes and increased that of immature DP CD4^low^CD8^+^ and SP CD4^low^ cells, compared to WT thymocytes. Therefore, the absence of TRPV1 impairs positive selection by reducing/delaying CD4^+^ thymocyte maturation. Moreover, a reduced percentage of mature CD8^+^ and CD4^+^ T cells was observed in PBMC and spleen from TRPV1 deficient mice. These findings are in agreement with previous reports demonstrating that an impaired thymic autophagy affects the distribution of mature SP CD4 and CD8 T cells in the periphery [51].

TRPV1, constitutively expressed in mouse and human CD4^+^ T cells, is a component of the TCR signaling complex. It is rapidly recruited to TCR clusters upon TCR stimulation in a Src-dependent manner, and contributes to CD4^+^ T cell activation. In response to TCR stimulation TRPV1 is rapidly tyrosine phosphorylated by the Lck kinase that regulates TRPV1 activity in CD4^+^ T cells [14]. Analysis of TCR signaling evidenced a diminished p38 and Jnk activation and NF-kB and NFAT-1 translocation to the nucleus in TRPV1 KO CD4^+^ T respect to WT cells [14]. Thus, the loss of TRPV1 resulted in a reduction of SP CD4 thymocytes, further supporting the recent findings on the intrinsic role of TRPV1 in the activation of and acquisition of pro-inflammatory properties by CD4^+^ T cells [14]. Thus, in WT thymocytes, TRPV1 activation, by inducing ER stress and proteasome dysfunction, triggers a compensatory autophagy that inhibits the UPR response and rescues DP^dull^ thymocytes from apoptosis. On the contrary, in TRPV1 KO thymocytes, characterized by defects in autophagy and proteasome system, the impaired TRPV1 signalling, stimulates the apoptosis of DP thymocytes and impairs thymocyte maturation induced by TCRβ/CD2 stimulation, leading to a decrease in the percentage of mature SP CD4^+^.

Thus, pharmacological modulation of TRPV1 expression and functions could represent a new therapeutic strategy for restraining or stimulating different inflammatory T cell responses.

## MATERIALS AND METHODS

### Animals

C57BL/6 (WT) and B6.129X1-Trpv1tm1Jul/J (TRPV1 KO) male mice were purchased from Harlan (Udine, Italy) and Jackson Laboratory (Bar Harbor, ME, USA), respectively, and housed as described previously [12]. Five TRPV1 KO and five WT animals (6-10 week-old) were sacrificed for each experiment in accordance with the U.S. National Institutes of Health’s “Guidelines for the Care and Use of Laboratory Animals”. Before and after sacrifice respectively, mice were weighted and thymi were measured by caliper without significant differences between WT and TRPV1 deficient mice.

### Cell preparation and culture conditions

Thymi were teased, and cellular debris were removed by washing. Thymocytes were isolated by centrifugation on a Lympholyte-M (Cederlane, Burlington, Canada) gradient and washed in RPMI 1640 medium supplemented with 5x 10^-5^ M β-mercaptoethanol, 2 mM glutamin, 100 U/ml penicillin, 100 µg/ml streptomycin, and 10% fetal calf serum (FCS). Cell purity (99%) was assessed by FACS analysis using anti-CD3 monoclonal antibody (mAb) (clone KT3) (10 μg/mL, Novus Biologicals, Littleton, CO, USA). Briefly, double positive (DP) CD4/CD8 cells were purified from 1x10^8^/ml total thymocytes by using the EasySep CD4 and CD8 purification kits (Stem Cell Technologies, Vancouver, Canada) according to the manufacturer’s instructions. The resultant cell population was enriched for CD4^+^CD8^+^ DP cells (approximately 97% of purified thymocytes).

To mimic positive selection, thymocytes were stimulated by anti-TCRβ mAb (clone: H57-597) plus anti-CD2 mAb (clone: 12-15) (10 μg/mL, Novus Biologicals) cross-linking. Briefly, thymocytes were cultured *in vitro* for 18 h in mAb-coated (stimulated thymocytes) or uncoated (non-stimulated thymocytes) Petri dishes (day 1) and then incubated for additional 12 h (day 2) in the absence of mAbs.

### Compounds

Rapamycin (an autophagy inducer inhibiting the mammalian target of rapamycin mTOR, 10 ng/ml), chloroquine (CQ, an autophagy inhibitor preventing the fusion between the autophagosome and lysosomes, 100 μM), capsaicin (CPS, TRPV1 agonist, 10 μM), capsazepine (CPZ, a synthetic, competitive and selective antagonist of TRPV1, 1 μM), 4-phenylbutyric acid (4-PBA, ER stress inhibitor, 5 mM), Ethylenediaminutesetetraacetic acid (EDTA), a calcium chelating agent, 5 mM), N-acetyl-L-cysteine (NAC, a free radical scavenger, 10 mM), trypan blue, dimethyl sulfoxide (DMSO) as well as the substrates for assaying the chymotrypsin-like (ChT-L), trypsin-like (T-L), peptidylglutamyl-peptide hydrolyzing (PGPH) and proteasome inhibitors, Z-Gly-Pro-Phe-Leu-CHO and lactacystin, were purchased from Sigma-Aldrich (St. Louis, MO, USA).

### Measurement of proteasome activity in cell lysates

Cells were washed in PBS and centrifuged at 1,600 g for 5 minutes. The pellet was resuspended in lysis buffer (20 mM Tris, pH 7.4, 250 mM sucrose, 1 mM EDTA and 5 mM β-mercaptoethanol) and passed through a 29-gauge needle at least ten times. Lysates were centrifuged at 12,000 x g for 15 minutes and the supernatants were stored at -80°C. Protein concentration was determined by the method of Bradford using bovine serum albumin (BSA) as standard.

Proteasome peptidase activity in cell lysates was determined with fluorogenic peptides: Suc-Leu-Leu-Val-Tyr-AMC was used for the chymotrypsin-like (ChT-L) activity, Z-Leu-Ser-Thr-Arg-AMC for the trypsin-like (T-L) activity and Z-Leu-Leu-Glu-AMC for the peptidylglutamyl-peptide hydrolyzing (PGPH) activity. The incubation mixture contained 1 μg of cell lysate, the appropriate substrate and 50 mM Tris-HCl pH 8.0, up to a final volume of 100 μL. Incubation was performed at 37 °C and after 60 minutes the fluorescence of the hydrolysed 7-amino-4-methyl-coumarin (AMC) was detected on a SpectraMax Gemini XPS microplate reader (λexc = 365 nm, λem = 449 nm). The 26S proteasome ChT-L activity was tested using Suc-Leu-Leu-Val-Tyr-AMC as substrate and a 50 mM Tris-HCl pH 8.0 buffer containing 10 mM MgCl_2_, 1 mM dithiothreitol, and 2 mM ATP. The effective 20S proteasome contribution to short peptide cleavage was evaluated performing control experiments using the specific proteasome inhibitors Z-Gly-Pro-Phe-Leu-CHO and lactacystin (5 μM in the reaction mixture) and then subtracting the obtained fluorescence values from the values obtained in cell lysates.

### Immunofluorescence and flow cytometry

Thymocytes from WT or TRPV1 KO mice were stained with anti-CD4-PE (clone: RM4-5) and/or anti-CD8-Cy5 (clone 53-6.7) mAb (1μl/1x10^6^ cells; BD Biosciences, Milan, Italy) and analyzed by a FACScan cytofluorimeter (BD Biosciences) using the CellQuest software. In some experiments, double immunofluorescence for CD4 and CD8 was performed on thymocyte from WT mice pre-treated for 1h with 4-PBA (5 mM), CQ (100 μM) or NAC (10 mM) and then treated with CPS (10 μM) for 2h. Moreover, the percentage of CD4 and CD8 positive cells was evaluated on thymocytes from WT or KO mice, un-stimulated or stimulated to mimic thymic positive selection. To investigate the role of TRPV1 in the activation of thymocytes, cells from WT mice were treated or not with CPS for 2 h, and then stained with anti-CD25-FITC (clone PC61) (1μl/1x10^6^ cells, Biolegend, San Diego, USA) or anti CD69-PE (clone H1.2F3) mAb (1μl/1x10^6^ cells, Biolegend).

### Western blot

Thymocytes from WT and KO mice were lysed as described previously [12]. Samples were separated on SDS-PAGE and transferred onto Hybond-C extra membranes (GE Healthcare, Milan, Italy). Membranes were incubated in 5% low-fat dry milk for 1 h and then overnight at 4°C in the primary antibodies solution: rabbit anti-p27 Kip1, anti-p62, anti-Grp94/gp96, anti-BiP/Grp78, anti-ERp57/Grp58, anti-Atf-4, anti-AMPK and anti-pAMPK (1:1000; Cell Signaling Technology, Danvers, MA, USA); rabbit anti-LC3 (2 μg/mL; Novus Biologicals); mouse anti-GAPDH (1:5000; Sigma-Aldrich). Thereafter, membranes were blotted with the respective horseradish peroxidase-conjugated anti-rabbit (1:2000; Cell Signaling Technology), anti-mouse (1:2000; Cell Signaling Technology) for 1 h. The detection was performed using the LiteAblot Plus or the LiteAblot Turbo (EuroClone, Milano, Italy) kits and densitometric analysis was carried out by evaluating three independent experiments by a ChemiDoc using Quantity One software (Bio-Rad, Hercules, CA, USA). Each sample was compared with its loading control (GAPDH) for quantification. In some experiments, thymocytes from WT mice were treated or not with CPS for different times. Moreover, thymocytes were pre-treated for 1 h with NAC, EDTA, CPZ, 4-PBA or CQ, before the addition of CPS for 2 h. In addition, TRPV1 KO thymocytes were treated for different times with Rapamycin.

### Annexin V and PI staining

Cell death was evaluated using Annexin V-fluorescein isothiocyanate (Annexin-FITC, BD Biosciences) and propidium iodide (PI, 2 μg/ ml, Sigma-Aldrich) followed by biparametric FACS analysis. Cells were stained with 5 μl of Annexin V-FITC and/or PI for 10 minutes at room temperature and washed once with binding buffer (10mM Hepes/NaOH pH 7.4, 140 mM NaCl, 2.5 mM CaCl_2_). The percentage of positive cells determined over 10,000 events was analyzed by a FACScan cytofluorimeter using the CellQuest software.

### RNA isolation, reverse transcription, and RT-PCR profiler array

Total RNA was extracted from thymocytes with the RNeasy mini kit (Qiagen, Valencia, CA, USA). Total RNA (1 μg) was subjected to reverse transcription using the ReactionReady first-strand cDNA kit (SuperArray Bioscience, Frederick, MD, USA). qRT-PCR was performed using the iQ5 Multicolor RT-PCR detection system (Bio-Rad), the RT2 real-time SYBR Green PCR mix, and the mouse Unfolded Protein Response PCR Array (SuperArray Bioscience), according to the manufacturer’s instructions.

### Statistical analysis

The statistical significance was determined by Student’s t test and by ANOVA with Bonferroni post-test. No statistically significant difference was found between untreated and vehicle (DMSO)-treated thymocytes (data not shown).

## SUPPLEMENTARY MATERIALS FIGURE



## Abbreviations

ChT-L: chymotrypsin-like
CPS: capsaicin
CPZ: capsazepine
CQ: chloroquine
DMSO: dimethyl sulfoxide
DP: double positive
EDTA: Ethylenediaminetetraacetic acid
ER: endoplasmic reticulum
ERAD: endoplasmic reticulum-associated degradation
NAC: N-acetyl-L-cysteine
PGPH: peptidylglutamyl-peptide hydrolyzing
PI: propidium iodide
ROS: reactive oxygen species
SP: single positive
T-L: trypsin-like
TRPV1: transient receptor potential vanilloid channel 1
UPR: unfolded protein response
UPS: ubiquitin-proteasome system
4-PBA: 4-Phenylbutyric acid
