# Supplementary material for: The TRPV1 ion channel regulates thymocyte differentiation by modulating autophagy and proteasome activity
Source: Oncotarget. 2017 Oct 11;8(53):90766–80. doi: 10.18632/oncotarget.21798 (PMC5710883; doi:10.18632/oncotarget.21798)
Supplement: Supplementary file 1 [file oncotarget-08-90766-s001.pdf]

The TRPV1 ion channel regulates thymocyte differentiation by modulating autophagy and proteasome activity

SUPPLEMENTARY MATERIALS

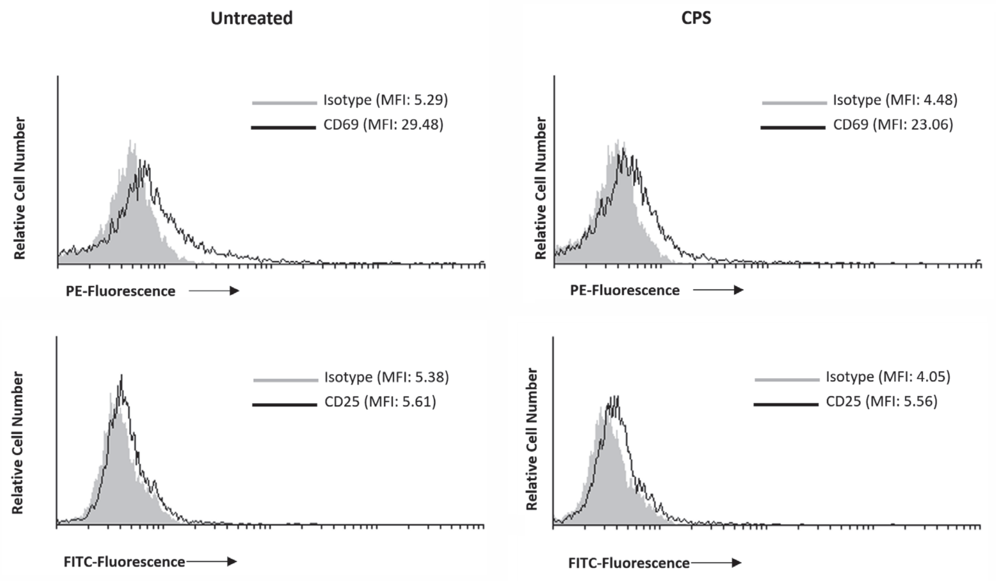

Supplementary Figure 1: CPS treatment does not stimulate the thymocyte expression of CD25 and CD69 activation markers. Immunofluorescence was performed on thymocytes from WT mice, treated or not with CPS for 2h. Cells were stained with CD25-FITC or CD69-PE and then analyzed by FACS. One representative out of three independent experiments is shown in each panel. MFI= Mean Fluorescent Intensity.
